# Factors Affecting ICU Stay and Length of Stay in the ICU in Patients with HELLP Syndrome in a Tertiary Referral Hospital

**DOI:** 10.1155/2022/3366879

**Published:** 2022-04-18

**Authors:** Elif Ağaçayak, Rezan Bugday, Nurullah Peker, Ugur Deger, Gönül Ölmez Kavak, Mehmet Siddik Evsen, Talip Gul

**Affiliations:** ^1^Department of Obstetrics and Gynecology, Dicle University School of Medicine, Diyarbakır, Turkey; ^2^Department of Obstetrics and Gynecology, Memorial Hospital, Diyarbakır, Turkey; ^3^Department of Anesthesia and Reanimation, Dicle University School of Medicine, Diyarbakır, Turkey

## Abstract

**Objective:**

The study aimed to compare patients with HELLP syndrome who require intensive care and who do not require intensive care and evaluate the factors affecting the length of stay in the intensive care unit.

**Methods:**

Patients were divided into two groups as follows: requiring intensive care (group 1) and not requiring intensive care (group 2). The data of both groups were compared in terms of demographic characteristics, transfusion amounts, length of stay in the intensive care unit, maternal complications, and mortality.

**Results:**

14032 births in a tertiary center between 2011 and 2018 were evaluated in this study. During the study period, 342 patients were diagnosed with HELLP, and 32 (9.4%) of these were followed up in the intensive care unit. The length of stay in the intensive care unit was determined as 8.1 (7.2) days on average. Fresh frozen plasma, erythrocyte suspension, apheresis, and random thrombocyte transfusion were observed to be significantly more in group 1 patients. In the regression analysis, the most effective factor was found to be erythrocyte suspension and the length of stay in the intensive care unit was significantly longer in patients who had erythrocyte suspension transfusion. The receiver operating characteristic curve showed that the area under the curve value for erythrocyte transfusion was 70.6%. When the cutoff value of erythrocyte suspension was 450 (95% CI: 365–681) ml, the sensitivity was 43.8% and the specificity was 91.6%.

**Conclusion:**

We think that physicians should be careful that maternal morbidity and mortality may increase as the need for erythrocyte suspension transfusion increases in patients with HELLP syndrome. Minimum transfusion to hemodynamically stable patients can be more suitable in terms of morbidity and mortality in managing patients with HELLP syndrome requiring erythrocyte suspension transfusion. Precautions and measures should be taken in this regard.

## 1. Introduction

HELLP syndrome is a syndrome first defined in 1982 by Weinstein, related to the complications seen in pregnancy or following the pregnancy, characterized by hemolysis, increased liver enzymes, and thrombocytopenia. It is seen in 1–10 of 1000 pregnancies and observed in 10–20% of serious preeclamptic pregnancies [1]. Although it is generally seen in the 3rd trimester of the pregnancy, it can also be seen in the first 48–72 hours following the delivery. 69% of the cases are observed during the antepartum, and 31% are observed during the postpartum period. Most of the postpartum cases can occur 48 hours after delivery [2]. Even though HELLP syndrome displays similarities with preeclampsia, hypertension and proteinuria are not observed in 10–20% of the cases [3].

The admission frequency of pregnant patients to intensive care units (ICUs) has been determined as 0.7–13.5 for every 1000 births [4]. Among the most common reasons for ICU admissions regarding obstetric patients are obstetric hemorrhages, hypertension disorders of the pregnancies, sepsis, amniotic fluid embolism, and pulmonary edema related to tocolysis. Complications related to HELLP syndrome are observed at 13–65% rates. The most commonly seen complications are blood transfusion requirement, common intravascular coagulation (DIC), acute renal failure (ARF), and acute respiratory distress syndrome (ARDS) [5]. In HELLP syndrome, maternal mortality rates are 2.5% [6]. The mortality rates for obstetric patients admitted to the intensive care unit vary between 1.3 and 20% [7]. As the length of stay in the ICU increases, maternal mortality increases as well [8]. Life-threatening, severe complications are seen in HELLP syndrome such as renal failure, pleural effusion, pulmonary edema, DIC, extended wound healing, endometritis, liver hematoma, retinal detachment, and multiple organ failure. Thus, it is one of the leading causes of intensive care stay indications in obstetric patients. In HELLP syndrome, two and more organ failures and/or ventilator requirements are some of the intensive care indications [5].

HELLP syndrome is the most complicated and difficult complication of pregnancy and is still not fully understood. Intensive care is required in approximately 10% of the patients. There are still serious debates in the literature about the course of HELLP syndrome and the factors that affect staying in intensive care in the HELLP syndrome. According to our working hypothesis, by determining the factors that affect staying in intensive care, we will be able to prevent early morbidities that may develop in intensive care. When we reviewed the literature, no studies were found on the factors affecting the length of stay in the intensive care unit, which is the most important factor affecting mortality in HELLP syndrome. Therefore, in our study, we aimed to compare patients with HELLP syndrome who need an ICU and those who do not and to evaluate the factors affecting the length of stay in the ICU. In this way, we aim to reduce mortality by minimizing the factors affecting the length of stay of intensive care.

## 2. Materials and Method

This study was carried out at a tertiary referral hospital, between January 2011 and December 2018. Between these dates, 14032 births took place in our hospital. Our hospital is a tertiary regional hospital and serves a region with the highest fertility and therefore highest natural population growth. It also accepted complicated cases from surrounding hospitals. Patients who were hospitalized with a preliminary diagnosis of HELLP at the same time patients who were delivered at the outer center and referred to our clinic with a preliminary diagnosis of HELLP were evaluated. Approval for the study was obtained from our university hospital local ethics committee (ethics committee date: 15.03.2019/No: 129).

Patients satisfying the following criteria were evaluated as having HELLP syndrome according to the Mississippi classification and were included in the study [9].

According to the Mississippi classification,

  Class 1. Thrombocyte ≤50,000 cells/microL, LDH >600 IU/L, and AST or ALT ≥70 IU/L
  Class 2. Thrombocyte >50,000–100,000 cells/microL, LDH >600 IU/L, and AST or ALT ≥70 IU/L
  Class 3. Thrombocyte >100,000, but ≤150,000 cells/microL and LDH >600 IU/L and AST or ALT ≥40 IU/L

Patients with pregnancies below 20 weeks, chronic liver disease, thrombotic thrombocytopenic purpura (TTP), acute fatty liver disease, hemolytic uremic syndrome (HUS), and gestational cholestasis were excluded from the study. Patients were divided into two groups as follows: requiring intensive care unit (group 1) and not requiring intensive care unit (group 2). Patients requiring intensive care unit were followed up in tertiary intensive care unit. Patients in need of intensive care unit were followed up in the same intensive care unit (reanimation clinic). All patients had the same intensive care standards. The group 1 patients requiring ICU were patients who needed antepartum and postpartum intubation and developing complications related to HELLP syndrome were monitored in the ICU. Coagulopathy and hemorrhage, ARF, infections, hepatic dysfunction, severe peripartum hemorrhage, DIC, sepsis, PRESS, and gastric bleeding were considered as complications. The patients who developed HELLP syndrome during the pregnancy were accepted as antepartum HELLP, and patients who developed it after giving birth were accepted as postpartum HELLP syndrome. The patients who were and were not treated with steroids (dexamethasone) to increase thrombocytes were recorded.

The data of both groups were compared in terms of age, gravida, parity, mode of delivery, gestational age at delivery, laboratory results, systolic and diastolic blood pressure, transfusion amounts, maternal complications, and mortality. Place of birth, multiple pregnancy, and newborn mortality were recorded. For the laboratory results of HELLP patients, the highest values at admission for parameters such as alanine aminotransferase (ALT), aspartate aminotransferase (AST), lactic dehydrogenase (LDH), bilirubin, international normalized ratio (INR), and the lowest values for platelet (PLT) were taken as a basis. Moreover, the highest values of urea and creatinine parameters from kidney function tests were taken as the basis. The protein value identified in the spot urine at the first referral of the patients was considered. Accordingly, 75 mg protein 1+, 150 mg protein 2++, and 300 mg protein 3+++ proteinuria were considered in the spot urine. The highest blood pressure (BP) values measured during the patients' referral or stay were taken as a basis. Bolus hydralazine, labetalol, or sublingual nifedipine dosages were performed to control serious hypertension (160/110 mmHg). Three intravenous dosages of 10 mg dexamethasone were administered at 12-hour intervals to increase the number of thrombocytes as soon as the real HELLP syndrome diagnosis was stated (before birth or after birth).

Transfusions performed preoperatively, intraoperatively, and postoperatively (erythrocyte suspension (ES), random thrombocyte, apheresis thrombocyte, and fresh frozen plasma (FFP) were evaluated as milliliter (ml)). Transfusions were approved according to the clinical condition of the patient, laboratory findings, and the time of the surgical procedure by the obstetrician and reanimation physician. In the case of tachycardia, tachypnea, syncope, faintness, and cerebral hypoxia symptoms, ES transfusion was performed according to the patients' hemoglobin (Hb) level. In most, hemodynamically stable medical and surgical patients, a hemoglobin level of 7-8 g/dl was considered. One unit of FFP was administered for every unit of ES. In our clinic, we perform thrombocyte transfusion when PLT is ≤20,000–25,000/*μ*L in vaginal delivery and when the PLT is <50,000/*μ*L in cesarean section and in those with class I HELLP syndrome [10]. We primarily prefer apheresis thrombocyte transfusion. However, in case of a problem with the apheresis device, we perform random thrombocyte transfusion. If the platelet value exceeds 100000 or tends to increase from the basal level, we stop platelet transfusion.

Termination of the pregnancy was performed in the following cases. 1—gestational age ≥37 weeks—delivery was performed without waiting for; 2—in the 34 weeks or older pregnancy weeks—delivery was performed following stabilization; and 3—before 34 weeks—delivery was performed within 48 hours after corticosteroid administration, if high blood pressure persists despite the use of antihypertensive drugs and if there is a risk of maternal and fetal complications. When the patient's hemodynamic and physiological condition is stabilized and the need for intensive patient monitoring was no longer necessary, she was discharged from the ICU and transferred to the obstetric clinic. Patients were transferred to the obstetric clinic consisted of patients who can be mobilized and self-care.

### 2.1. Statistical Analyses

Statistical analyses were carried out using the SPSS 21 program. Descriptive information was given using mean, standard deviation, numbers, and percentages. For the comparison of patients who required and did not require intensive care, the Kolmogorov–Smirnov test was used to evaluate whether they fit the normal distribution. The chi-square test was used for the categorical data evaluation. Student's *t*-test was used in the comparison of continuous variables fitting normal distribution. The Mann–Whitney *U* test was used for binary comparisons of nonparametric tests to compare continuous variables that do not fit the normal distribution. Spearman's correlation and linear regression analysis were performed to determine the factors affecting the length of stay in the ICU. Sensitivity, specificity, and area under the curve (AUC) calculations were conducted with the ROC curve analysis.

## 3. Results

14032 births in a tertiary center between 2011 and 2018 were evaluated in this study. During the study period, 342 patients were diagnosed with HELLP, and 32 (9.4%) of these were followed up in the intensive care unit. 36 (10.5%) of the patients were antepartum, and 29 (8.5%) patients were referred to our clinic from the periphery with the preliminary diagnosis of postpartum HELLP. 277 (81%) patients were patients who applied directly to our center. According to the Mississippi classification, 50 (14.7%) of the patients were class 1, 164 (47.9%) of them were class 2, and 128 (37.4%) of them were class 3. 47 (36%) of class 3 patients had partial HELLP syndrome. They were patients with thrombocyte levels above 150,000 but with hemolysis and elevated liver enzymes (excluding hepatitis, gestational cholestasis, and cholelithiasis). 81.2% of the patients who stayed in ICU were in Mississippi classes 1 and 2.

**Figure 1:**
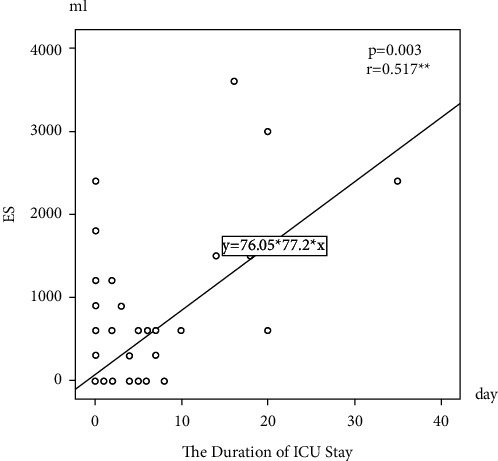
Relationship chart between erythrocyte suspension transfusion amount and length of stay in the intensive care unit.

**Figure 2:**
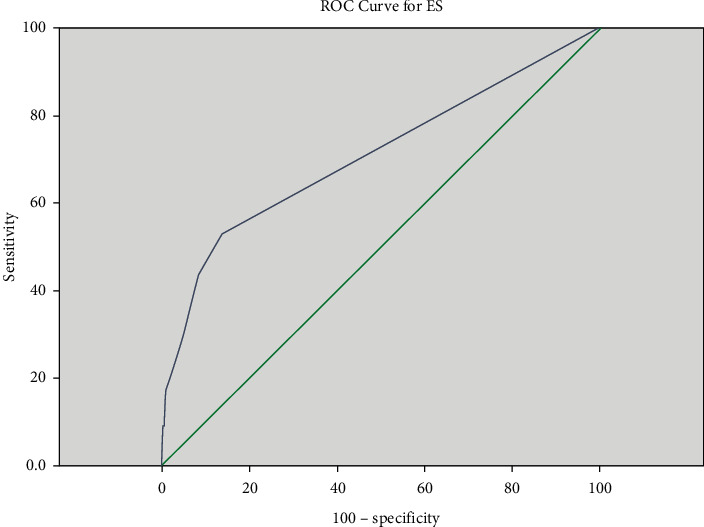
Receiver operating characteristic curve for erythrocyte suspension transfusion amount.

174 (50.9%) of the patients were multiparous when the demographic data were evaluated. Gestational age was between 28 and 36 weeks in the 210 (61.4%) of the patients. C-section delivery rates were 286 (83.7%). Hypertension and proteinuria were identified in 307 (89.8%) of the patients, whereas thrombocytopenia was identified in 295 (86.3%) of the patients. Blood transfusion was performed on 93 (27.2%) of the patients. 2 (0.6%) of the patient required a relaparotomy because of post-C-section bleeding. Postpartum HELLP syndrome was observed in 84 (24.6%) patients. 3 (0.8%) patients in need of antepartum and postpartum intubation and mechanical ventilation, 8 (2.3%) patients with ARF but improved without dialysis, 1 (0.3%) patient who developed postpartum cardiomyopathy and needed inotropic support , 2 (0.6%) patients who developed subarachnoid bleeding, 2 (0.6%) patients who had a serious peripartum hemorrhage and underwent relaparotomy, 4 (1.1%) patients diagnosed with DIC, 2 (0.6%) patients diagnosed with sepsis, 10 (2.9%) patients diagnosed with PRESS, and 1 (0.3%) patient diagnosed with gastric bleeding were admitted to ICU. Our maternal mortality rate was 2 (0.6%) (Table 1). Patients who developed mortality were in class 1 according to the Mississippi classification. However, a significant difference in terms of the developing maternal complications was not observed between the 3 groups. 32 (9.4%) of the patients with HELLP syndrome were monitored in ICU. The length of stay in the ICU was determined as 8.1 (7.2) days on average. The maternal mortality rate for patients with HELLP syndrome who were admitted to ICU was found as 6.25%. The demographic data of the patients staying (group 1) and not staying (group 2) in the intensive care were compared. AST, LDH, bilirubin, and creatinine were significantly higher in group 1 patients, and PLT was significantly lower. Transfusion was performed significantly more in group 1 patients. It was observed that TDP, ES, apheresis, and random platelet transfusion were significantly higher in group 1 patients (Table 2). When the factors that affect the length of stay in the ICU were evaluated, a positive correlation was found between age, AST, LDH, creatinine, ES, and FFP (Table 3). In the regression analysis, the independent predictor factor was found to be erythrocyte suspension and the length of stay in the ICU was significantly higher in patients who had erythrocyte suspension transfusion (Table 4, Figure 1). The receiver operating characteristic curve showed that the AUC value for erythrocyte transfusion was 70.6%. When the cutoff value of erythrocyte suspension was 450 (95% CI: 365–681) ml, the sensitivity was 43.8% and the specificity was 91.6% (Figure 2). A significant effect of giving birth outside of the center, postpartum HELLP syndrome, multiple pregnancies, C-section deliveries, and dexamethasone performed after birth to increase platelets, during the intensive care stay, was not observed.

## 4. Discussion

In this study, patients diagnosed with HELLP syndrome were evaluated. Demographic and laboratory parameters of patients staying in intensive care unit and factors affecting the length of stay in the intensive care in patients staying in intensive care unit were investigated. According to the results, it was determined that as erythrocyte suspension transfusion increases, the length of stay in the ICU is extended.

HELLP syndrome is seen in 0.1–1% of all pregnant women and 1-2% of patients with serious preeclampsia and eclampsia. It constitutes 5.5% of obstetric patients staying in ICU [11]. In this study, it was determined that HELLP syndrome constitutes 2.4% of all births, 7.9% of patients with preeclampsia and eclampsia, and 8.3% of patients staying in ICU due to obstetric causes. Compared with the literature, we attribute our high rates to the fact that our hospital is a tertiary referral hospital and that high-risk pregnancies are directed to our clinic.

In our study, the mean age of occurrence of HELLP syndrome was 30.1 (7.0), and the mean age of occurrence in patients staying in the intensive care unit was 27.3 (7.2). In accordance with the literature, advanced maternal age was not a risk factor for ICU in our study [12]. However, an advanced maternal age significantly increases the length of stay in the ICU. For this reason, it might be necessary to be more careful and quick, especially when approaching mothers with an advanced age and HELLP syndrome. Although the age factor is a factor affecting the length of stay in the ICU, it was determined that the age factor was not an independent predictive factor when the confounding factors were adjusted and evaluated. We think that this is due to the fact that the age distribution of our patients is not normal. At the same time, we think that age is not an independent predictive factor since the maternal risks may increase in a physiological pregnancy with increasing age.

Unlike preeclampsia, nulliparity is not a risk factor for HELLP syndrome, and half of the affected patients or more are multiparous [13, 14]. In this study, where the results were consistent with the literature, most of the patients were multiparous. Gravida and parity were not found as effective risk factors on intensive care stay and the length of stay in the ICU.

Hypertension and proteinuria are seen in approximately 85% of HELLP syndrome. In 10–20% of patients with HELLP syndrome, neither of these findings might be observed [15]. In this study, where the results were consistent with the literature, it was identified that hypertension and proteinuria were not effective factors on ICU stay and the length of stay in the ICU.

In a study evaluating HELLP syndrome and complicated pregnancies, 70% of the symptoms occurred before birth, and 80% of these cases develop before the 37th pregnancy week [16]. In this study, similar results to the literature were identified and it was observed that the conditions such as gestational week and postpartum development of HELLP are not factors affecting ICU stay and the length of stay in the ICU.

In the literature, it is reported that the mode of delivery in HELLP syndrome is 75% of C-sections. Additionally, since 91% of the patients staying in ICU gave birth with a C-section, it is thought that C-section might be a risk factor for intensive care [17]. In a literature study evaluating whether multiple pregnancies were a risk factor in terms of HELLP syndrome, it was seen in approximately 1.9% of multiple pregnancies and it was determined that the present and following pregnancies were a risk factor for HELLP syndrome [18]. In this study, multiple pregnancy rates were consistent with the literature, and it was observed that C-section rates were relatively higher. We attribute our higher C-section rates compared with the literature to the fact that most of our patients were class 1 and class 2 in the Mississippi classification and our hospital was a tertiary referred center. Moreover, during the transfer, the need for C-section increases due to the maternal and fetal risks of the patients. Although our C-section rates were found to be high, it was identified that both C-section and multiple pregnancies were not risk factors for ICU stay and the length of stay in the ICU. Although our cesarean rates were high, cesarean section was not determined as a risk factor for the length of stay in the ICU and ICU stay. We think that the reason for this is that our hospital is a fully equipped hospital, and therefore, our cesarean section complications are low. At the same time, another reason may be that we have an experienced team in approaching multiple pregnancy.

In a study evaluating patients with HELLP syndrome in the literature, it was determined that high ALT, AST, LDH, bilirubin, and low platelets are significantly effective factors on maternal mortality [19]. In another study, the increase in bilirubin and LDH was determined to be a risk factor regarding the development of maternal complication [20]. In this study, similar results were obtained regarding patients staying in ICU. It was observed that AST, LDH, and creatinine were factors that especially affect the length of stay in the ICU. Thus, we think that patients who have high AST and LDH and creatinine levels during the initial admission should be closely monitored. In the first evaluation, we think that the reason why the increase in liver and kidney function tests is a factor that increases the length of stay and stays in intensive care may be due to liver and kidney failure that may develop in the later stages. Although AST, LDH, and creatinine were factors affecting the length of stay in the ICU, it was determined that these parameters were not independent predictive factors when the confounding factors were adjusted. We think that this is due to the fact that our data are independent of each other and their distribution is not normal. At the same time, changes in biochemical parameters in a physiological pregnancy may be a factor affecting this result. If we could use the ensemble model as the regression model, it would be more appropriate for the current study results [21].

Complications related to HELLP syndrome are observed in 13–65%. The most commonly seen complication is a blood transfusion requirement, hemorrhage, DIC, ABY, and ARDS [15, 22]. In another study, transfusion requirements in HELLP syndrome were highlighted to be significantly higher compared with other hypertensive disorders in pregnancies [23, 24]. In a study in the literature evaluating the complications developed in patients staying in the intensive care unit, it was found that maternal complications were significantly higher, especially in Mississippi class 1 [25]. In this study, it was identified that 27.2% of patients required blood transfusion, and relaparotomy was performed on 0.6% due to bleeding. It was found that transfusions were performed significantly more in patients staying in intensive care. It was observed that especially ES and FFP transfusions significantly increased the length of stay in the ICU. The reason for our morbidity rates to be lower compared with the literature may be due to the fact that our clinic is a tertiary reference hospital, so we encounter many types of patients and have gained experience in the management of these cases. A significant difference was not observed between the patients staying and not staying in intensive care in terms of maternal complications. A significant difference was not observed between antepartum HELLP and postpartum HELLP in terms of maternal complications and intensive care stay. It was seen that Mississippi class 1 patients stayed significantly longer in intensive care. However, a significant difference was not found according to the Mississippi classification in terms of maternal complications.

Maternal mortality rates are 3.5–24.2% in the HELLP syndrome [25]. The causes of maternal mortality related to HELLP syndrome are renal failure, DIC, pulmonary and cerebral edema, ablatio placentae, hepatic hemorrhage, and hypovolemic shock [26, 27]. Also, it is reported that a long time stay in the intensive care unit is one of the most important factors that affect maternal mortality [28]. The average length of stay in the ICU of patients with HELLP syndrome is 3.47 (3.16) days [11]. In this study, the average length of stay in the ICU for patients with HELLP syndrome was determined as 8.1 (7.2) days. Two of our patients who stayed in the intensive care passed away. Our maternal mortality rate was observed as 0.6%. One of the patients who developed maternal mortality had a DIC diagnosis, and the other had a sepsis diagnosis. Although we had a longer length of stay in ICU compared with the literature, it was seen that our maternal mortality was lower. We attribute this to the fact that our hospital is a fully equipped tertiary regional hospital. Our hospital is a center with a very high birth rate, where HELLP syndrome cases in our region are accepted and have an experienced team.

As the length of hospitalization in intensive care takes longer, infections complications and associated costs increase in intensive care [29]. In our study, it was found that erythrocyte suspension transfusion was the most important predictor factor that independently affects the length of stay in the ICU. Transfusions of 450 cc and above were found to be the most important factor affecting the length of stay in the ICU. In this study, 25% of the patients who were staying in intensive care had anemia. Although anemia is not among the factors that affect the length of stay in the ICU, the fact that ES transfusion is one of the most important factors that affect the length of stay in the ICU suggests the risks created by blood transfusion. There are infections, hemolysis, allergies, febrile reactions, and electrolyte imbalances related to blood transfusion [30]. An average of 1800 (2545) cc erythrocyte transfusion was performed on our two patients who developed maternal mortality. For most patients, we recommend a limiting transfusion strategy rather than an excessive transfusion strategy. This means giving less blood, transferring at a lower hemoglobin level, and targeting a lower hemoglobin level. On the other hand, for patients who require more than 450 cc of ES transfusion, it should be considered that their intensive care stay might be extended and additional protective precautions should be taken for the patients in advance. One of the most important factors that increase the need for blood transfusion is hemolysis in patients with HELLP syndrome. When considered as an independent clinical picture, similar to preeclampsia, abnormal placentation accompanies HELLP syndrome, but unlike preeclampsia, excessive hepatic inflammation and overactivation of the coagulation system play a role in the pathogenesis. Hemolysis increases over time [31]. The problem in the coagulation system should be tackled in the first place, and if we have a predefinition of HELLP, coagulation parameters should be kept in balance to prevent quickly bleeding. Re-evaluation of clinical files in patients with the increasing need for blood transfusion may provide the necessary precautions for future patients.

The limitation of our study is that it is not a clinical and prospective study. It is an observational, retrospective study. Another limitation of our study is that we did not use the ensemble model to adjust for confounding factors, since our dependent variables were not discrete. Nonetheless, the advantage of this study is that it is the first evaluation of intensive care stay and the length of stay in the intensive care unit for patients with HELLP syndrome. To the best of our knowledge, no studies on this subject have been found in the literature. The number of cases was high enough to find differences between groups, and also, the methodology was comprehensive and repeated. These were also some other advantages of this study.

## 5. Conclusion

It could be noted that the most effective independent parameter affecting intensive care stay and length of stay in the intensive care of patients with HELLP syndrome is ES transfusion. Thus, we think that physicians should be careful that maternal morbidity and mortality may increase as the need for ES transfusion increases in patients with HELLP syndrome. Minimum transfusion to hemodynamically stable patients can be more suitable in terms of morbidity and mortality in managing patients with HELLP syndrome requiring ES transfusion. Precautions and measures should be taken in this regard.

## Figures and Tables

**Table 1:** Evaluation of demographic and laboratory findings in the patients with HELLP syndrome.

		*n*	Percent (%)
Parity	Nulliparous	168	49.1
	Multiparous	174	50.9

Gestational age at delivery (week)	0–22	18	5.3
	23–27	56	16.3
	28–36	210	61.4
	37–40	58	17

Mode of delivery	Normal vaginal delivery	56	16.3
	Cesarean section	286	83.7

Hypertension	Yes	307	89.8
	No	35	10.2

Thrombocytopenia	Yes	295	86.3
	No	47	13.7

Anemia	Yes	50	14.6
	No	292	85.4

Proteinuria	+1 (75 mg)	98	28.6
	+2 (150 mg)	160	46.7
	+3 (500 mg)	49	14.3
	No	35	10.2

Mississippi classification	Class 1	50	14.6
	Class 2	164	48.0
	Class 3	128	37.4

Maternal complication	ARF	8	2.3
	GIB	1	0.3
	PRESS	10	2.9
	DIC	4	1.1
	Sepsis	2	0.6
	Postpartum cardiomyopathy	1	0.3
	SAB	2	0.6
	Total	28	8.1

Blood transfusion	Yes	93	27.2
	No	249	72.8

Type of blood transfusion	Fresh frozen plasma (cc)	43	24.3
	Erythrocyte suspension (cc)	59	33.3
	Random suspension (cc)	18	10.2
	Apheresis platelet suspension (cc)	57	32.2

Dexamethasone treatment	Yes	252	73.6
	No	90	26.4
Reason of relaparotomy	Intraabdominal major bleeding	2	0.6

Postpartum HELLP	Yes	84	24.6
	No	258	75.4

Place of birth	Delivery outside of the center	65	19
	Delivery in the center	277	81

Multiple pregnancy	Yes	10	2.9
	No	342	97.1

Maternal mortality	Yes	2	0.6
	No	340	99.4

Newborn mortality	Yes	18	5.2
	No	324	94.8

Data are presented as percent. ARF: acute renal failure, GIB: gastrointestinal bleeding, PRESS: posterior reversible encephalopathy syndrome, DIC: disseminated intravascular coagulation, SAB: subarachnoid bleeding.

**Table 2:** Comparison of demographic data in patients staying and not staying in ICU.

		Patients requiring ICU Group 1 **n** **=** **32 (9.4%)**	Patients not requiring ICU Group 2 **n** **=** **310 (90.6%)**	*p*
Age		27.3 (7.2)	30.4 (6.9)	0.792
Gravidity		3.3 (3.2)	3.9 (3.4)	0.129
Parity		2.1 (2.4)	2.4 (2.5)	0.378
Gestational age at delivery (week)		31.1 (4.0)	32.2 (4.0)	0.159
Hematocrit (%)		34.6 (6.0)	36.2 (5.6)	0.140
Hemoglobin (g/dl)		11.5 (2.0)	12.2 (2.3)	0.155
Platelet (K/uL)		73.6 (46.1)	106.3 (66.7)	**0.001**
ALT (U/L)		262.2 (225.6)	153.6 (166.5)	**0.002**
AST (U/L)		530.7 (384.9)	225.6 (252.4)	**<0.001**
LDH (IU/L)		1481.8 (642.8)	805.3 (489.4)	**<0.001**
Total bilirubin (mg/dl)		3.4 (2.8)	2.9 (2.3)	**<0.001**
Urea (mg/dl)		36.3 (27.1)	28.1 (13.9)	0.078
Creatinine (mg/dl)		1.3 (6.6)	1.2 (1.1)	**0.001**
Proteinuria (mg)		120 (68)	120 (60)	0.735
INR		1.8 (2.8)	1.1 (0.4)	0.074
Systolic blood pressure (mmHg)		156.4 (28.3)	155.4 (19.9)	0.561
Diastolic blood pressure (mmHg)		98.4 (18.9)	96.7 (11.9)	0.320
Blood transfusion	Yes	20 (62.5%)	73 (23.5%)	**<0.001**
	No	12 (37.5%)	237 (76.5%)	
Fresh frozen plasma (ml)		483.9 (814.9)	65.0 (215.2)	**<0.001**
Erythrocyte suspension (ml)		690.7 (924.6)	84.4 (260)	**<0.001**
Random suspension (ml)		583.3 (1100.7)	89.9 (356.0)	**<0.001**
Apheresis platelet suspension (ml)		612.5 (973.8)	84.9 (324.5)	**<0.001**
Dexamethasone treatment	Yes	26 (81.3%)	226 (72.9%)	0.144
	No	6 (18.7%)	84 (27.1%)	
Mississippi classification	Class 1	13 (40.6%)	38 (12.2%)	**<0.001**
	Class 2	13 (40.6%)	151 (48.7%)	
	Class 3	6 (18.8%)	121 (39.1%)	
Postpartum HELLP	Yes	10 (31.3%)	72 (23.2%)	0.317
	No	22 (68.7%)	238 (76.8%)	
Mode of delivery	Normal vaginal delivery	6 (18.75%)	50 (16.1%)	0.486
	Cesarean section	26 (81.25%)	260 (83.9)	
Multiple pregnancy	Yes	1 (3.1%)	9 (2.9%)	0.594
	No	31 (96.9%)	301 (97.1%)	
Place of birth	Delivery outside of the center	10 (31.3%)	55 (17.7%)	0.066
	Delivery in the center	22 (68.7%)	255 (82.3%)	
Maternal complication	ARF	6 (18.7%)	2 (0.6%)	0.074
	GIB	1 (3.1%)	0	
	PRESS	6 (18.7%)	4 (1.3%)	
	DIC	4 (12.5%)	0	
	Sepsis	2 (6.25%)	0	
	Postpartum cardiomyopathy	2 (6.25%)	0	
	SAB	1 (3.1%)	0	
Newborn mortality	Yes	1 (3.1%)	17 (5.5%)	0.568
	No	31 (96.9%)	293 (94.5%)	

Data are presented as mean ± SD or number (percent). Mann–Whitney *U* test, chi-square test, *p* < 0.05 statistically significant (in bold). ICU: intensive care unit, ALT: alanine aminotransferase, AST: aspartate aminotransferase, LDH: lactate dehydrogenase, INR: international normalized ratio, ARF: acute renal failure, GIB: gastrointestinal bleeding, PRESS: posterior reversible encephalopathy syndrome, DIC: disseminated intravascular coagulation, AB: subarachnoid bleeding.

**Table 3:** Spearman's correlation analysis of factors affecting the length of stay in the ICU.

**n: 32**	*r*	*p*
Age	0.385^*∗*^	0.030
AST (U/L)	0.405^*∗∗*^	0.022
LDH (IU/L)	0.501^*∗∗*^	0.004
Creatinine (mg/dl)	0.505^*∗∗*^	0.003
Erythrocyte suspension (ml)	0.517^*∗∗*^	0.003
Fresh frozen plasma (ml)	0.537^*∗∗*^	0.002

ICU: intensive care unit, AST: aspartate aminotransferase, LDH: lactate dehydrogenase. Spearman's correlation analysis.

**Table 4:** Regression analysis in terms of factors affecting the length of stay in the ICU.

**n: 32**	BETA	*t*	*p*	**95%** CI
Constant: **3.427**				
Age	0.026	0.193	0.848	−0.250–0.301
AST (U/L)	0.007	2.024	0.055	0.000–0.014
LDH (IU/L)	−0.003	−1.261	0.220	−0.007–0.002
Creatinine (mg/dl)	0.144	0.165	0.870	−1.667–1.955
Erythrocyte suspension (ml)	**0.010**	**3.786**	**0.001**	**0.004**–**0.015**
Fresh frozen plasma (ml)	−0.005	−1.718	0.099	−0.011–0.001

ICU: intensive care unit, AST: aspartate aminotransferase, LDH: lactate dehydrogenase. Linear regression analysis.

## Data Availability

The data have been protected by the local ethics committee of Dicle University Faculty of Medicine in terms of patient rights. The readers can contact Assoc. Dr. Elif Agacayak (drelifagacayak@gmail.com) for any data request.

## Supplementary Materials

Supplementary Materials A linear regression model met the assumptions of homogeneity and normality of the residuals. SPSS graphic results showing normality and homoscedasticity in linear regression analysis results were uploaded as supplemental files.
