# Supplementary material for: Factors Affecting ICU Stay and Length of Stay in the ICU in Patients with HELLP Syndrome in a Tertiary Referral Hospital
Source: Int J Hypertens. 2022 Apr 18;2022:3366879. doi: 10.1155/2022/3366879 (PMC9038419; doi:10.1155/2022/3366879)
Supplement: Supplementary Materials — A linear regression model met the assumptions of homogeneity and normality of the residuals. SPSS graphic results showing normality and homoscedasticity in linear regression analysis results were uploaded as supplemental files. [file 3366879.f1.docx]

**Assumptions of Homogeneity and Normality of the Residuals in the Linear Regression Analysis**


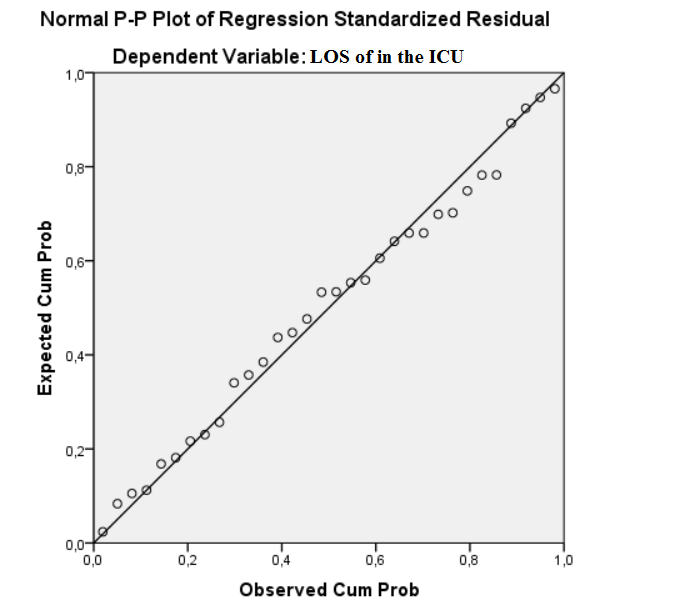


Figure showing that errors of the estimations are normally distributed.


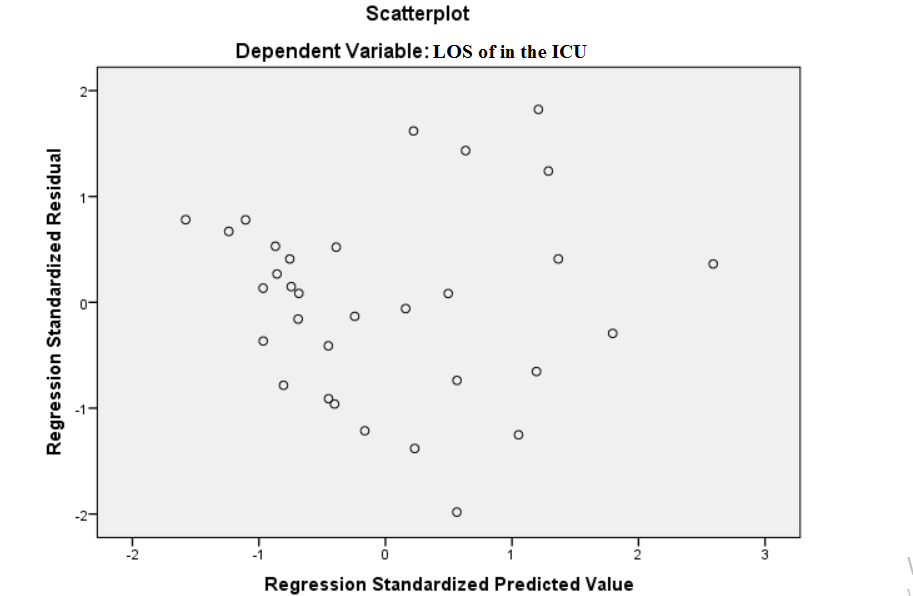


Figure showing homoscedasticity : There is covariance between independent variables and dependent variable


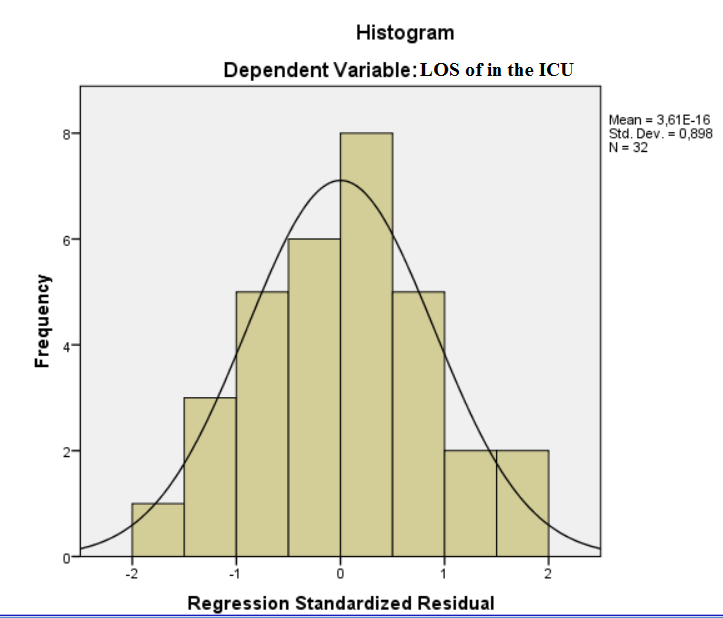


Histogram image showing the distribution of errors
